# Incidence of and risk factors for medical care interruption in people living with HIV in recent years

**DOI:** 10.1371/journal.pone.0213526

**Published:** 2019-03-13

**Authors:** Anna Lucie Fournier, Yazdan Yazdanpanah, Renaud Verdon, Sylvie Lariven, Claude Mackoumbou-Nkouka, Bao-Chau Phung, Emmanuelle Papot, Jean-Jacques Parienti, Roland Landman, Karen Champenois

**Affiliations:** 1 IAME, UMR1137, INSERM, Paris Diderot University, Sorbonne Paris Cité, Paris, France; 2 Infectious and Tropical Diseases Department, Normandie Univ, UNICAEN, Normandie University Hospital, Caen, France; 3 Infectious and Tropical Diseases Department, Bichat-Claude Bernard University Hospital, AP-HP, Paris, France; 4 GRAM 2.0, EA2656, Normandie Univ, UNICAEN, Normandie University Hospital, Caen, France; The Ohio State University, UNITED STATES

## Abstract

**Objectives:**

With HIV treatment as a prevention strategy, retention in care remains a key for sustained viral suppression. We sought to identify HIV-infected patients at risk for medical care interruption (MCI) in a high-income country.

**Methods:**

The HIV-infected patients enrolled had to attend the clinic at least twice between January 2010 and October 2014 and were followed up until May 2016. MCI was defined as patients not seeking care in or outside the clinic for at least 18 months, regardless of whether they returned to care after the interruption. The association between MCI and sociodemographic, clinical, and immuno-virological characteristics at HIV diagnosis and during follow-up was assessed using Cox models.

**Results:**

The incidence rate of MCI was 2.5 per 100 persons-years (95% confidence interval [CI] = 2.3–2.7). MCI was more likely in patients who accessed care >6 months after diagnosis (hazard ratio [HR] = 1.30, 95% CI = 1.10–1.54 *vs*. ≤6 months) or did not report a primary care physician (HR = 2.40; 95% CI = 2.03–2.84). MCI was less likely in patients born in sub-Saharan Africa (HR = 0.75, 95% CI = 0.62–0.91 *vs*. born in France). During follow-up, the risk of MCI increased when the last CD4 count was ≤350 (HR = 2.85, 95% CI = 2.02–4.04 *vs*. >500 cells/mm^3^) and when the patient was not on antiretroviral therapy (HR = 3.67, 95% CI = 2.90–4.66).

**Conclusions:**

The incidence of MCI is low in this hospital that serves a large proportion of migrants. Low or no recorded CD4 counts for a medical visit could alert of a higher risk of MCI, even more in patients who accessed HIV care late or did not report a primary care physician.

## Introduction

Antiretroviral treatment (ART) allows people living with HIV (PLHIV) to achieve a life expectancy close to that of the general population [1,2]. The international CASCADE cohort showed a dramatic decrease in mortality rate due to HIV from 40.8 for 1,000 person-years in 1996 to 6.1 in 2004 thanks to the advent of highly active antiretroviral therapy [3]. A French study found AIDS as the cause of death in 2.2 of 1,000 HIV-infected patients during follow-up in 2010 [4]. Now that HIV has become a chronic disease with lifelong treatment, in addition to HIV diagnosis, one of the challenges is to maintain, enhance, and facilitate retention of HIV-infected patients in the care system.

Continuing engagement in care is the key to achieving 95% viral suppression in treated HIV patients by 2025 the third target of UNAIDS in fast-tracking the end of the AIDS epidemic [3,5–7]. At a population level, retention in care and ART adherence, in sustaining an undetectable viral load, limit onwards HIV transmissions, in other words enhance the treatment as prevention (TasP) concept [6,8–11]. At an individual level, medical care interruption (MCI) leads to loss of immunity and complications of HIV infection [10]. In a 2009 French study, HIV-infected patients who interrupted care for more than one year and then came back into care had a risk of death 5-fold higher than regular follow-up PLHIV [7].

Most previous studies highlighted factors collected at the time of HIV diagnosis that were associated with an MCI [12,13], even a few in Northern countries [10,14]. To our knowledge, no study has assessed the risk factors for MCI during the follow-up period. Finding those events that could precipitate patients into an MCI could help physicians spot a difficult time period during follow-up and prevent an MCI. Our aims were to estimate the annual incidence rate of MCI and to identify the risk factors, at the time of diagnosis and during follow-up, of MCI in HIV-infected patients enrolled in a large clinical cohort in Paris from 2010–2016.

## Materials and methods

### Description of the cohort

Patients were part of the HIV clinical cohort of the Infectious Diseases Department of Bichat University Hospital in Paris, France. In addition to inpatient and outpatient services, a desk is dedicated to medical visits without an appointment, and a multidisciplinary support team, including social workers and community NGO staff, works with physicians to support patients in their medical care and administrative procedures. Enrolled patients were ≥18 years old and had attended the clinic at least twice between January 2010 and October 2014. All the patients enrolled in this study gave their written informed consent to have their medical chart concerning HIV data (sociodemographic, clinical, immunological, virological, and therapeutic) recorded in Nadis, the electronic medical record system designed for medical follow-up of HIV-infected patients. The Nadis database has been declared to the French “commission national informatique et liberté” (CNIL, www.dataids.org Fedialis Medica, Marly Le Roi, France, CNIL number: 1171457 May 24th 2006) [15]. No further ethic approval was necessary according to the French regulation. Electronic medical records were prospectively filled in by medical or paramedical staff at each clinic visit of patients. If an appointment was missed, the patient or the primary care physician if the patient could not be reached was contacting by the infectious diseases referent for information; patient outcomes were recorded as medical summaries in the medical charts. Patients were followed until May 2016.

### Definition of patients with medical care interruption

MCI was deemed to have occurred if patients did not seek care in the infectious diseases clinic or at an outside facility for at least 18 months, regardless of whether they came back into care after the interruption. We chose an 18-month period rather than the 12-month period used in some other studies [13,14,16–18], because French HIV guidelines have moved towards fewer laboratory tests and visits to healthcare centers per year of PLHIV care. The 2013 French guidelines recommend administration of ART to each PLHIV whatever the CD4 count; a once-a-year visit to an infectious disease specialist for patients on ART whose HIV infection has been under control (undetectable viral load and CD4 T cell count >500 cells/mm3) for one year without comorbidity; and a clinical and immuno-virological check-up at least every 6 months, which can be performed by the primary care physician [17].

When the calculated time between each visit to the infectious disease clinic exceeded 18 months, the patient’s medical record was systematically reviewed. For each case of MCI, we actively searched for any medical contacts or blood tests after the last known visit by reading the medical summaries completed by the attending physicians. Thus, we were able to know if the patients sought care in another department or another hospital, have been followed by another physician outside the hospital, have moved to another region/country, or had died during the period without a visit to the clinic. If a patient came back to care after a presumed MCI with a viral load under 50 copies/mL, we considered that an indication that ART had not been interrupted and that MCI had not occurred, assuming follow-up elsewhere during this period [10].

### Variables

We considered sociodemographic, clinical, immunological, virological, and therapeutic data at HIV diagnosis and during follow-up. We assessed time-dependent variables such as age (≤30 years, 31–45 years, and >45 years), comorbidity (included surgical, medical and psychiatric events), the occurrence of an AIDS indicator condition and viral hepatitis coinfection status, CD4 count, viral load, and ART prescription at each medical visit. If no data were found between day 14 before and day 14 after the medical visit, the time dependent variable(s) were considered to be missing data.

**Fig 1:**
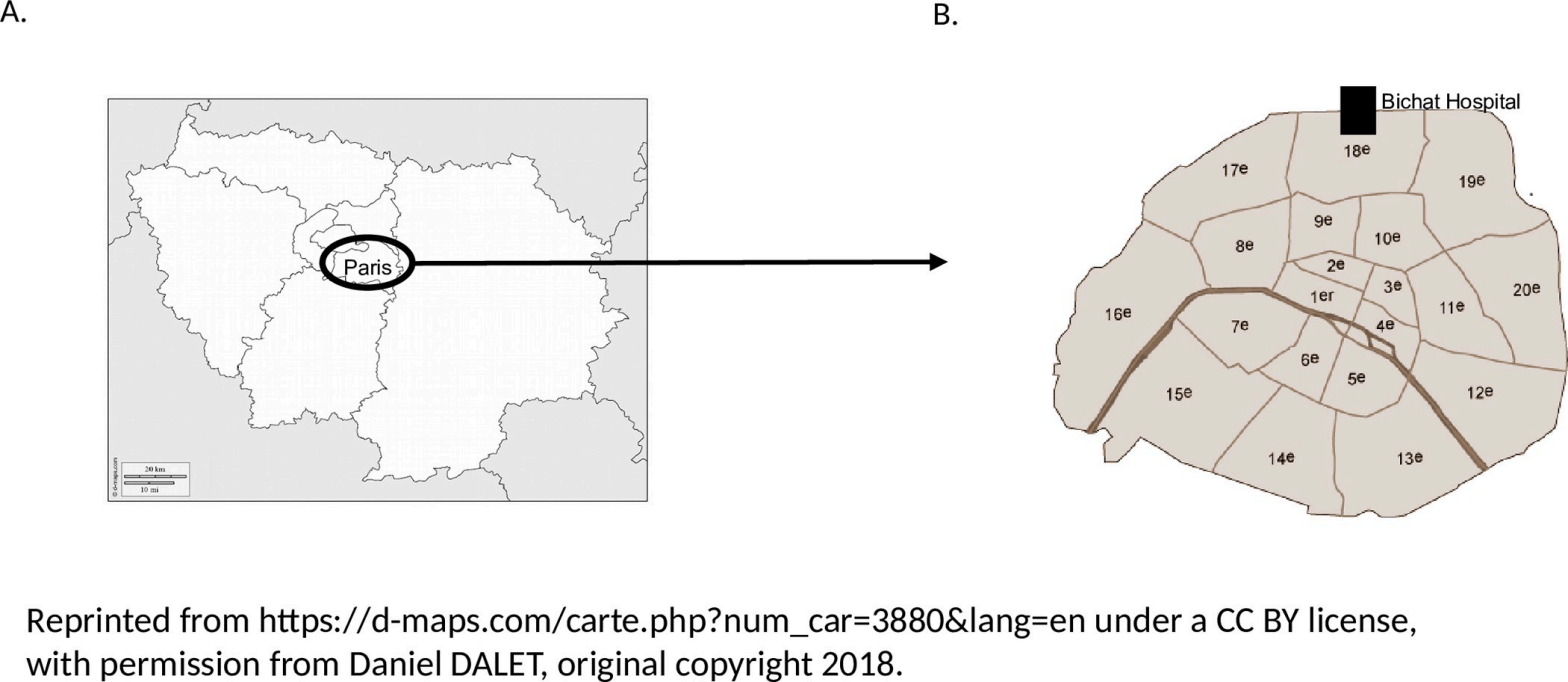
Geographical area of patients’ residence and poverty rates in Ile-de-France. Among the 4,796 patients participating in the study, 285 lived outside the Ile-de-France region, and no mailing addresses were found for 115 patients. The other patients lived in the Ile-de-France area and were distributed as follows: A. Ile-de-France region, excluding Paris: 2,234 patients where the poverty rate in 2016 was 14%. B. Paris city: 1,610 patients where the poverty rate was 16%; including the 18th arrondissement of Paris: 552 patients where the poverty rate was 24% [22,23].

We collected the last mailing address of each patient to document, to determine their distance from the hospital (and assess any difficulties in accessing the facility) and the level of deprivation of the neighborhood [19–21]. Deprivation may influence access and retention in care. The hospital is located in the 18th arrondissement (district) of Paris, one of the poorest areas in the Ile-de-France region, where Paris is situated (Fig 1) [22,23]. Thus, we classified people as living in the 18th arrondissement in Paris, in Paris as a whole (minus the 18th), in the Ile-de-France region (except Paris), and elsewhere or without an available postal code.

### Statistical analyses

We estimated the incidence rate of MCI as the number of patients who had at least one MCI of ≥18 months, divided by the number of person-years at risk of MCI. Individuals were considered at risk of MCI from the date of enrollment in the study (January 1, 2010 or the date of first visit in Bichat if they initiated care there at a later date) to their last visit plus 6 months or not later than May 31, 2016, the end-point of the analysis. People who interrupted care were considered from their last visit plus 6 months, when a clinical and immunovirological check-up should have been performed according to French guidelines [17]. Deceased participants were censored at the date of death, if known, or at their last visit plus 3 months. An exact Poisson distribution was used to estimate 95% confidence intervals (CIs). To compare our results with those of other studies, we also estimated the incidence rate of MCI ≥12 months.

The associations between MCI and sociodemographic, clinical and immuno-virological characteristics at HIV diagnosis and during follow-up were assessed using a Cox model. The proportional hazard assumption was checked using Schoenfeld residuals. The variables which did not respect the proportional hazard assumption were excluded from the model. The variables associated with MCI with a p-value <0.20 in the univariate analysis were entered into the multivariate model. A backward stepwise regression analysis was used to build the final multivariate model. Variables were considered significant if the Wald test p-value was <0.05. Interactions between variables were systematically checked. Statistical analyses were performed using STATA 14 (Stata Corporation, College Station, TX, USA).

## Results

Between January 2010 and October 2014, 4,796 patients had at least two encounters at the Bichat Hospital. Sociodemographic, clinical, biological, and therapeutic characteristics are shown in Table 1. Most were men (63%). Participants were born in France (38%), sub-Saharan Africa (40%), or elsewhere (20%). Heterosexual men and women transmission group represented almost half (48%) of participants: 30% were men who have sex with men (MSM), and 5% were people who inject drugs (PWID).

**Table 1:** Characteristics associated with Medical Care Interruption (MCI) at the time of HIV diagnosis of patients seeking HIV care in the infectious diseases department of the Bichat hospital, Paris, between January 2010 and May 2016.

	All N = 4,796	Regular follow-up (N = 4,179)	MCI ≥18 months (N = 617)
**Age at HIV diagnosis (years)**			
≤30	1,855 (39%)	1,603 (38%)	252 (41%)
[31–45]	2,275 (47%)	2,004 (48%)	271 (44%)
>45	666 (14%)	572 (14%)	94 (15%)
**Year**^**a**^ **of enrollment in care**			
<2002	2,041 (43%)	1,800 (43%)	241 (39%)
[2002–2006[	930 (19%)	797 (19%)	133 (22%)
[2006–2011[	1,064 (22%)	925 (22%)	139 (23%)
[2011–2013[	571 (12%)	494 (12%)	77 (12%)
≥2013	190 (4%)	163 (4%)	27 (4%)
**Sex**			
Male	3,041 (63%)	2,633 (63%)	408 (66%)
Female	1,755 (37%)	1,546 (37%)	209 (34%)
**Country of birth**			
France	1,824 (38%)	1,568 (38%)	256 (41%)
Sub-Saharan Africa	1,916 (40%)	1,695 (40%)	221 (36%)
Other countries or unknown^**b**^	1,056 (22%)	916 (22%)	140 (23%)
**Residence**			
Ile-de-France	2,234 (46%)	1,978 (47%)	256 (41%)
Paris (except the 18th arr.)	1,610 (34%)	1,393 (33%)	217 (36%)
18th arrondissement of Paris	552 (12%)	471 (11%)	81 (13%)
Other areas	285 (6%)	248 (7%)	37 (6%)
No available postal code	115 (2%)	89 (2%)	26 (4%)
**Primary care physician**^**c**^			
Yes	3,648 (76%)	2,359 (56%)	283 (46%)
No	1,148 (24%)	1,820 (44%)	334 (54%)
**HIV transmission group**			
Men who have sex with men	1,462 (31%)	1,290 (31%)	172 (28%)
Heterosexual men and women	2,314 (48%)	2,032 (49%)	282 (46%)
People who inject drugs	257 (5%)	218 (5%)	39 (6%)
Other or no data available^d^	763 (16%)	639 (15%)	124 (20%)
**AIDS**^**e**^ **at HIV diagnosis**			
Yes	367 (8%)	326 (8%)	41 (7%)
No	4,429 (92%)	3,853 (92%)	576 (93%)
**Hepatitis B coinfection**			
Yes	269 (6%)	240 (6%)	29 (5%)
No	4,527 (94%)	3,939 (94%)	588 (95%)
**Hepatitis C coinfection**			
Yes	269 (6%)	243 (6%)	26 (4%)
No	4,527 (94%)	3,936 (94%)	591 (96%)
**HIV viral load at enrollment (copies/mL)**^**f**^			
≤1,000	1,294 (27%)	1,114 (27%)	180 (29%)
1,001–10,000	736 (16%)	625 (15%)	111 (18%)
10,001–100,000	1,492 (31%)	1,331 (32%)	161 (26%)
>100,000	1,225 (26%)	1,086 (26%)	139 (23%)
**CD4 count at enrollment (cells/mm**^**3**^**)**			
≤350	2,348 (49%)	2,060 (49%)	288 (47%)
>350	2,448 (51%)	2,119 (51%)	329 (53%)
**Time period before first visit**^**g**^			
≤6 months	3,165 (66%)	2,882 (69%)	368 (60%)
>6 months	1,631 (34%)	1,382 (31%)	249 (40%)
**Time between first visit and first ART**^**h**^			
≤12 months	2,293 (49%)	2,054 (49%)	239 (39%)
>12 months	2,359 (51%)	2,053 (49%)	306 (50%)

MCI: Medical Care Interruption

^**a**^**Year of enrolment** are presented in classes corresponding to years of different HIV treatment initiation guidelines. In France, it was recommended to initiate ART under 200 CD4/mm3 in 2002, <350 CD4/mm^**3**^ in 2006; <500 CD4/mm^**3**^ in 2011 and regardless of CD4 count in 2013.

^**b**^The country of birth was missing for 73 participants

^**c**^Primary care physician declared by the patient, written in the computer file, to whom medical records from the clinic are sent

^**d**^The HIV transmission group information was missing for 587 participants

^**e**^AIDS: acquired immune deficiency syndrome defined by using the CDC classification

^**f**^The viral load data were missing for 49 participants

^**g**^Time between HIV diagnosis and first medical visit in or outside the clinic

^**h**^ART: antiretroviral therapy, prescribed for 4,652 patients by the end of the study period

Sixty-two percent of participants were diagnosed before 2006. At the time of HIV diagnosis, 8% of participants had an AIDS-defining event, 6% an HBV and 6% an HCV co-infection. The median HIV viral load at enrollment in care was 18,900 copies/mL [interquartile range, IQR = 608–105,160]. The median CD4 count at enrollment was 354 cells/mm3 [IQR = 191–539]. The median time between HIV diagnosis and the first medical contact for HIV was 1.3 months [IQR = 0.2–20] and under 6 months for 66% of individuals. The median time between the first medical visit for HIV and ART initiation was 12.0 months [IQR = 1.9–58.4]. A mailing address was reported by 98% of patients (Fig 1). A referring primary care physician was declared by 76% of patients.

### Identification of medical care interruption

**Fig 2:**
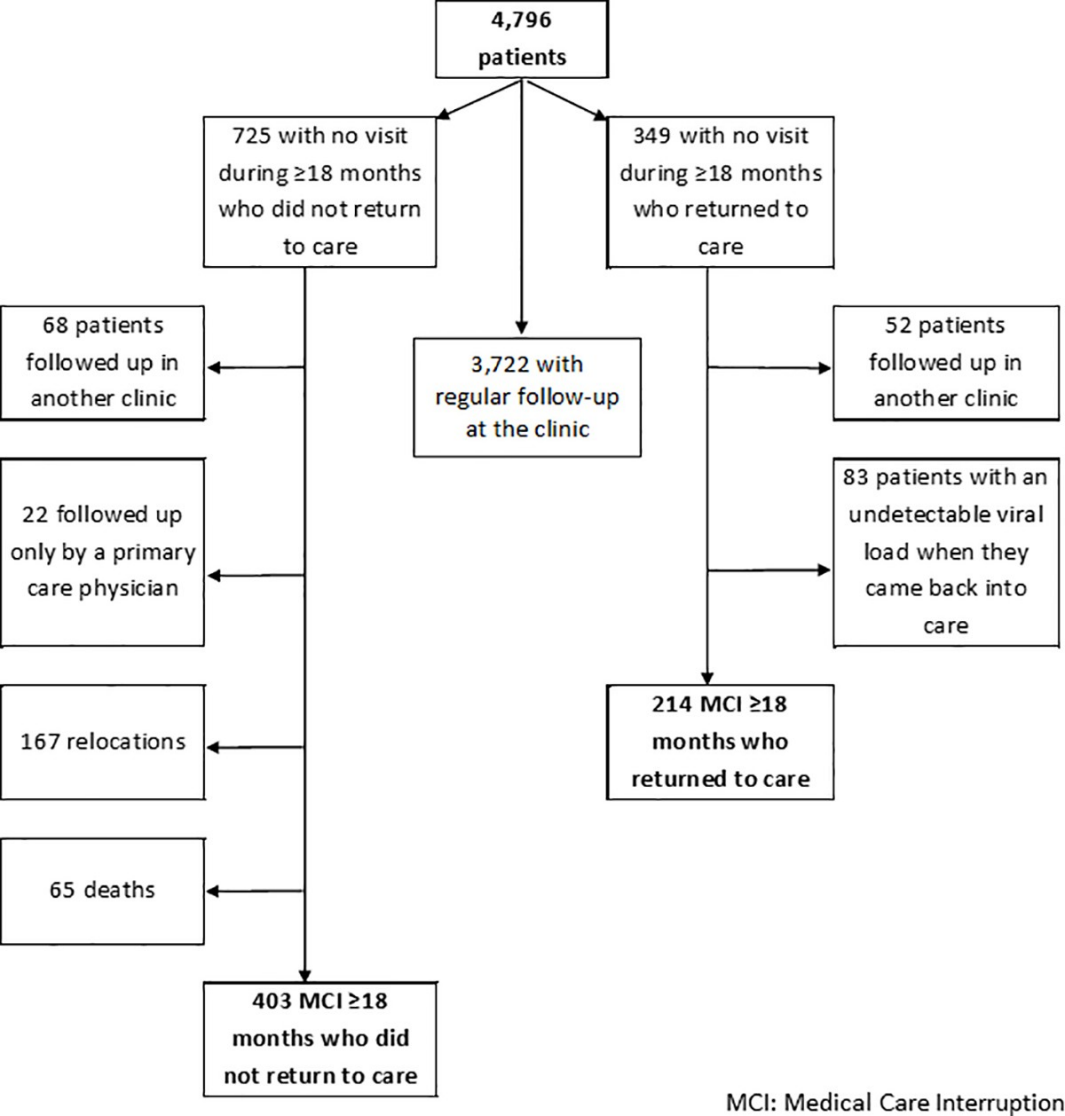
Future of HIV-infected patients with no health care visit for at least 18 months.

Among the 4,796 patients included, we found 725 patients with a priori no visits in our clinic during at least 18 months and no news at the end-point of the study. Active research highlighted that 68 of them had medical information from another department recorded in their medical records, 65 had died, 167 had moved to another region, and 22 had a primary care physician conduct follow- up for their HIV infection by a primary care physician. Finally, 403 patients were considered to have had at least one MCI and never returned to care (Fig 2).

We also identified 349 patients with no visits to our clinic during at least 18 months but who returned to care during the study period. Among these 349 patients, medical record evaluations showed that 52 patients had medical information recorded during the interruption period; 83 patients came back into care with an undetectable HIV viral load and were considered to have been received follow-up care elsewhere during this time period. Thus, 214 patients had at least one MCI and returned to care.

Overall, 617 of 4,796 (13%) patients had at least one MCI during follow-up and 214 (35%) of them returned to care. Overall, 617 of 4,796 (13%) patients had at least one MCI during follow-up and 214 (35%) of them came back into care.

### Incidence of MCI

The 4,796 patients accounted for 24,215 person-years at risk of MCI ≥18 months. We estimated an incidence rate of having at least one MCI ≥18 months of 2.5 per 100 person-years [95% CI = 2.3–2.7]. The median duration of follow-up in our study for people who interrupted care was 27.7 months [IQR = 10.5–43.9] *vs*. 71.3 months [IQR = 49.0–74.7] for those who did not; p<0.001. Considering MCI ≥12 months, we found 874 events among 27,312 person-years, i.e. an incidence rate of 3.2 [95% CI = 3.0–3.5].

### Factors associated with having at least one MCI ≥18 months

Factors associated with having at least one MCI in the univariate analysis are shown in Table 2. A lower risk of MCI was found for people aged between 31 and 45 years at the time of HIV diagnosis (HR = 0.83, 95% CI = 0.70–0.99 *vs*. ≤30 years old). People born in sub-Saharan Africa were less likely to have an MCI than were those born in France (HR = 0.79, 95% CI = 0.65–0.95). People living in the 18^th^ arrondissement of Paris and in Paris as a whole were more likely at risk of MCI than people living in Ile-de-France (HR = 1.39, 95% CI = 1.08–1.79 and HR = 1.22, 95% CI = 1.02–1.47, respectively). A high risk of MCI was found when any postal code was provided by the patient (HR = 2.08, 95% CI = 1.38–3.15). People who stated that they did not have a primary care physician were more likely to have an MCI (HR = 2.52, 95% CI = 2.14–2.98). A low viral load at the time of HIV diagnosis was associated with a higher risk of MCI (HR = 1.25, 95% CI = 1.00–1.58 for ≤1,000 and HR = 1.36; 95% CI = 1.05–1.76 for 1,001–10,000 *vs*. >100,000 copies/mL). Patients were more likely to have an MCI when they accessed care more than 6 months after the HIV diagnosis (HR = 1.34, 95% CI = 1.14–1.58 *vs*. presenting within the first 6 months) and when the first ART was prescribed more than one year after the first HIV care visit (HR = 1.18, 95% CI = 0.99–1.41 *vs*. less than one year).

**Table 2:** Univariate and multivariate analysis of factors associated with Medical Care Interruption (MCI) at the time of HIV diagnosis and during follow-up of patients seeking HIV Care in the infectious diseases department of Bichat hospital, Paris, between January 2010 and May 2016.

	Hazard ratio* (95% CI)	p-value*	Adjusted hazard ratio** (95% CI)	p-value**
**Fixed variables**				
**Age at HIV diagnosis (years)**				NS
≤30	1	-		
[31–45]	0.83 (0.70–0.99)	0.04		
>45	1.02 (0.80–1,30)	0.86		
**Sex**				NS
Male	1.17 (0.99–1.39)	0.06		
Female	1	-		
**Country of birth**				
France	1	-	1	-
Sub-Saharan Africa	0.79 (0.65–0.95)	0.01	0.75 (0.62–0.91)	0.004
Other countries or data unknown^a^	0.94 (0.76–1.16)	0.55	0.90 (0.73–1.12)	0.35
**Residence**				
Ile-de-France	1	-	1	-
Paris (except the 18th arr.)	1.22 (1.02–1.47)	0.03	1.20 (0.99–1.46)	0 .06
18th arrondissement of Paris	1.39 (1.08–1.79)	0.01	1.38 (1.07–1.78)	0.01
Other area	1.15 (0.81–1.65)	0.44	1.13 (0.79–1.63)	0.50
No available postal code	2.08 (1.38–3.15)	<0.001	2.09 (1.38–3.18)	0.001
**Primary care physician**^**b**^				
Yes	1	-	1	-
No	2.40 (2.14–2.98)	<0.001	2.40 (2.03–2.84)	<0.001
**HIV transmission group**				NS
Men who have sex with men	1	-		
Heterosexual men and women	0.96 (0.79–1.17)	0.71		
People who inject drugs	1.24 (0.87–1.78)	0.24		
Other or no data available^c^	1.35 (1.07–1.72)	0.01		
**AIDS**^d^ **at HIV diagnosis**				NS
Yes	0.79 (0.57–1.1)	0.15		
No	1	-		
**Hepatitis B coinfection**				
Yes	0.81 (0.56–1.19)	0.27		
No	1	-		
**Hepatitis C coinfection**				NS
Yes	0.69 (0.45–1.04)	0.06		
No	1	-		
**HIV viral load at enrollment (copies/mL)**^**e**^				NS
≤1,000	1.25 (1.00–1.58)	0.05		
1,001–10,000	1.36 (1.05–1.76)	0.02		
10,001–100,000	0.96 (0.76–1.21)	0.73		
>100,000	1	-		
**CD4 count at enrollment (cells/mm**^**3**^**)**				NS
≤350	1	-		
>350	1.13 (0.96–1.33)	0.15		
**Time period before first visit**^**f**^				
≤6 months	1	-	1	-
>6 months	1.34 (1.14–1.58)	<0.001	1.30 (1.10–1.54)	0.003
**Time between first visit and first ART**^**g**^				NS
≤12 months	1	-		
>12 months	1.18 (0.99–1.41)	0.05		
**Time-dependent variables**				
**Age (years)**				
≤30	1	-		
[31–45]	0.87 (0.62–1.21)	0.41		
>45	0.75 (0.54–1.05)	0.10		
**CD4 count (cells/mm**^**3**^**)**				
>500	1	-	1	-
[351–500]	1.39 (0.95–2.04)	0.09	1.42 (1.02–2.08)	0.04
≤350	2.54 (1.80–3.59)	<0.001	2.85 (2.02–4.04)	<0.001
Missing data	4.06 (3.2–5.13)	<0.001	3.87 (3.06–4.89)	<0.001
**Antiretroviral therapy**				
Yes	1	-	1	-
No	3.52 (2.79–4.45)	<0.001	3.67 (2.90–4.66)	<0.001
**AIDS**				
Yes	0.91 (0.75–1.11)	0.37		
No	1	-		

MCI: Medical Care Interruption

*Hazard ratios and p-values of univariate Cox models carried out for 4,796 patients

**Hazard ratios and p-values of the final multivariate Cox model (backward procedure) carried out for 4,796 patients. NS: non-significant (p>0.05)

^**a**^The country of birth was missing for 73 participants

^**b**^Primary care physician declared by the patient, written in the computer file, to whom medical records from the clinic are sent

^**c**^The HIV transmission group information was missing for 587 participants

^**d**^AIDS: acquired immune deficiency syndrome defined by using the CDC classification

^**e**^The viral load data were missing for 49 participants

^**f**^Time between HIV diagnosis and first medical visit in or outside the clinic

^**g**^ART: antiretroviral therapy, prescribed for 4,652 patients by the end of the study period

During follow-up, compared to patients with a CD4 count >500 cells/mm3, the risk of MCI increased when the CD4 count was ≤350 cells/mm3 (HR = 2.54, 95% CI = 1.80–3.59) and when no CD4 count was found in the patients’ medical files during the month around the visit (HR = 4.06, 95% CI = 3.20–5.13). People who were not on ART were more likely to have an MCI (HR = 3.52, 95% CI = 2.79–4.45). Comorbidities at HIV diagnosis, health events, and viral load during follow-up did not respect the proportional hazard assumption and were not entered into the multivariate Cox model. Age at HIV diagnosis, sex, AIDS status, hepatitis C coinfection, CD4 count at enrollment and time before first ART were not found to be independently associated with MCI (Table 2). The final multivariate model was carried out using 109,192 visits. At baseline, risk factors found to be independently associated with MCI were living in the 18th arrondissement in Paris (HR = 1.38, 95% CI = 1.07–1.78) or not having an available address (HR = 2.09, 95% CI = 1.38–3.18) vs. living in Ile-de-France outside of Paris, not indicating a primary care physician (HR = 2.40, 95% CI = 2.03–2.84), and >6 months between diagnosis and the first HIV care visit (HR = 1.30, 95% CI = 1.10–1.54). Factors associated with a lower likelihood of having an MCI were being born in sub-Saharan Africa or in a country other than France (HR = 0.75, 95% CI = 0.62–0.91 and HR = 0.79, 95% CI = 0.63–0.99, respectively vs. born in France). During follow-up, the identified risk factors were having a CD4 count ≤500 cells/mm3 with a higher risk of MCI for lower CD4 levels (HR = 1.42, 95% CI = 1.02–2.08 when 350< CD4 ≤500; HR = 2.85, 95% CI = 2.02–4.04 when ≤350 vs. >500) or missing data for CD4 levels (HR = 3.87, 95% CI = 3.06–4.89), and not being on ART (HR = 3.67, 95% CI = 2.90–4.66).

## Discussion

Among the 4,796 patients who received follow-up for their HIV infection in this French clinical cohort from 2010 to 2016, the incidence rate of having at least one MCI ≥18 months was estimated at 2.5 per 100 person-years. Patients had an increasing risk of MCI if they lived in the neighborhood surrounding the hospital, were born in France, accessed care more than 6 months after the HIV diagnosis or reported no primary care physician. The risk was also higher if, during follow-up, they did not receive ART and had a CD4 count that was either ≤500 cells/mm3 or was not recorded in the electronic medical file.

The incidence of MCI estimated in our study was lower than those estimated in other studies. In France, the estimated incidence rates of MCI are between 3.5 and 17.2 per 100 person-years [10,14,16,24]. The highest rate was in French Guiana, which may be explained by a lack of care settings, precariousness, and HIV stigmatization [24]. Our estimates were lower than the lowest estimates reported to date in France, even if we use the same time definition of MCI (12 months, 3.2 per 100 person-years). Several reasons may explain this difference. First, unlike other studies, we considered that patients had no MCI if they had any contact with care in or outside the hospital vs. only medical visits in the follow-up facility. Second, the comprehensive care management for PLHIV that has been implemented by the Infectious Diseases Department of Bichat Hospital, might facilitate patient retention in care; there are mainly medical visits without appointment, but proactive methods such as text messages or phone calls are used as appointment reminders. Third, the active search for information on patients first assumed to be lost to follow-up reduced the incidence of MCI to a more realistic level, and was the major strength of our study. Because of this approach, we obtained information for 43% of the 1,074 patients first identified with MCI.

The analysis of variables at the time of diagnosis helped characterize patients at risk of MCI. Patients living around the hospital were more likely to have an MCI than those living outside of Paris, suggesting that MCI might be associated with economic deprivation rather than difficulty accessing the hospital. A recent study in Philadelphia found a link between shorter distance to medical care and poor retention [25].

In contrast with all other studies on the topic [14,16,18,24], we found that migrants from sub-Saharan Africa were more likely to be retained in care than patients born in France. For example, Ndiaye et al. [14] found that African patients were at higher risk of interrupting care and not returning (HR = 1.81; 95% CI = 1.16–2.80), than HIV-infected patients born in France. A possible explanation for these opposing results stem from the fact that the Bichat Hospital Infectious Diseases Department has for years treated a large number of immigrants living in the 18th district of Paris or nearby. A high proportion of patients followed for HIV there were born in sub-Saharan Africa, which is much higher than representation in other centers (40% versus 13% in the French HIV national cohort FHDH [18]). The presence of community NGOs in the HIV department and African patients as peer educators to guide and support other HIV patients may have helped improve retention in care for this population at risk of MCI in the past. Moreover, the French care system provides universal access to care whatever the person's legal status, which limits discrimination regarding origins. Since 1998, migrants have been able to ask for state medical care “AME” and permanence of access to care “PASS” (Art. L. 711-7-1 Public health code) [26]. The role of social workers is very important to help foreigners navigate administrative procedures for obtaining State medical care.

Patients who reported a primary care referent had a lower risk of MCI, and may be an indication of care and concern about their health. Furthermore, an older French study also showed that having a primary care physician is a key to retention in care [14]. The collaboration between infectious diseases and primary care physicians seems crucial for the continuity of care. The automatic sharing of reports between doctors could improve the monitoring of patients [27]. Follow-up of patients at primary care may also reduce some structural barriers related to the hospital, such as transportation access, physician availability, wait time and appointment scheduling [28,29].

We evaluated risk factors of MCI in order to spot these events in the daily follow-up and act to prevent them. Time-dependent ART status was found to be associated with MCI. The treatment represents a link between the care system and the patient [1]. In the UK, people not on ART had a 5-fold higher risk of interrupting care than those on ART [30]. In agreement with other studies, we found an association between low CD4 counts during follow-up and MCI [13,14,18]. Low CD4 counts just before an MCI could be explained, at least in part, by poor adherence to treatment and care. Moreover, we found that patients seen at the clinic without an available CD4 count record had a 4-fold higher risk of MCI than those with CD4 counts >500 cells/mm3. We may assume that decreasing CD4 counts or a lack of CD4 measures could be a warning sign of MCI risk.

Our study presented some limitations. First, although we conducted active research of the patients in follow-up, there may remain a misclassification bias of patients wrongly assumed to be lost to follow up. Indeed, we could not consult the incarceration and National Death Index registries. This may have resulted in overestimation of the incidence rate of MCI by wrongly considering these people as having interrupted care. However, in another French study conducted from 1997 to 2006, Ndiaye et al. identified only 2 out of 1,007 patients initially considered lost to follow-up in the National Death Index registry [14]. Second, because we worked with a hospital database, we had no detailed information on the social context (deprivation, life priority needs) or legal status of patients who could be reasons for an MCI. A recent Italian study found that it was more the legal status (in particular undocumented status) than the country of birth that was associated with the risk of MCI [31]. Third, because of the specific recruitment of the hospital situated in a very deprived area with a high proportion of migrants in particular those born in sub-Saharan Africa, we cannot extrapolate our results to other settings. Fourth, the growing literature on rapid ART initiation after HIV diagnosis showed that the retention in care is improved by an early treatment initiation [32]. In our study, we could expect to find an association between an increasing time to treatment and MCI. However, this cross-sectional study evaluated the whole cohort during a time period, and not only newly diagnosed patients, assuming the context and medical practices were important. In return, we observed heterogeneity in terms of years of HIV care initiation. Time to initiate HIV treatment evolved with ART guidelines throughout the period and may explain that the median time between HIV diagnosis and ART initiation was so high (12 months).

In conclusion, we observed a low incidence of MCI in a hospital with a large proportion of very deprived people and of migrants from high HIV-prevalence countries. Low CD4 counts or no recorded CD4 measures for a medical visit could alert the physician of a current high risk of MCI, even more so in patients who accessed care late or did not report a primary care physician. Our findings may help clinicians identify HIV-infected patients who are at risk of interrupting care and to initiate appropriate interventions earlier for a better retention in care. Further interventions with multidisciplinary HIV care management, involving primary care physicians, could be evaluated to help retain HIV-infected patients in care.
